# The Influence of Bioactive Oxylipins from Marine Diatoms on Invertebrate Reproduction and Development

**DOI:** 10.3390/md7030367

**Published:** 2009-08-21

**Authors:** Gary S. Caldwell

**Affiliations:** School of Marine Science and Technology, Newcastle University, Ridley Building, Claremont Road, Newcastle upon Tyne, NE1 7RU, England, UK; E-Mail:Gary.Caldwell@ncl.ac.uk; Tel.: +44 (0) 191 222 6660; Fax: +44 (0) 191 222 7891

**Keywords:** polyunsaturated aldehydes, reproductive toxicity, apoptosis, developmental stability, teratogen

## Abstract

Diatoms are one of the main primary producers in aquatic ecosystems and occupy a vital link in the transfer of photosynthetically-fixed carbon through aquatic food webs. Diatoms produce an array of biologically-active metabolites, many of which have been attributed as a form of chemical defence and may offer potential as candidate marine drugs. Of considerable interest are molecules belonging to the oxylipin family which are broadly disruptive to reproductive and developmental processes. The range of reproductive impacts includes; oocyte maturation; sperm motility; fertilization; embryogenesis and larval competence. Much of the observed bioactivity may be ascribed to disruption of intracellular calcium signalling, induction of cytoskeletal instability and promotion of apoptotic pathways. From an ecological perspective, the primary interest in diatom-oxylipins is in relation to the potential impact on energy flow in planktonic systems whereby the reproductive success of copepods (the main grazers of diatoms) is compromised. Much data exists providing evidence for and against diatom reproductive effects; however detailed knowledge of the physiological and molecular processes involved remains poor. This paper provides a review of the current state of knowledge of the mechanistic impacts of diatom-oxylipins on marine invertebrate reproduction and development.

## 1. Introduction to Diatom Chemical Defence

Diatoms are among the most important photoautotrophic organisms, driving food web dynamics in some of the most productive marine systems, particularly in areas of nutrient upwelling. The classical view of aquatic food webs generally considers diatoms as passive participants in energy transfer, being heavily grazed by their predators and exhibiting little or no anti-grazing capacity [1,2]. Based on this paradigm, little consideration has been given to the possible evolution of diatom defensive strategies, in particular chemical defences, and the consequent role of such defences in regulating energy flow. Diatoms do posses defensive capabilities, most notably in the form of mechanical protection conferred by the silica frustule [3]. It is now apparent that complex and highly evolved chemical defences may also be in operation in many species [4–6]; however the topic of diatom chemical ecology is not without controversy [7–12]. Somewhat paradoxically, other autotrophs, primarily dinoflagellates and cyanobacteria, are renowned for producing highly toxic biomolecules [13], some of which have been linked to reproductive failures. The ingestion of toxic dinoflagellates by female copepods has been suggested detrimental for nauplii hatching success [14]. Yan *et al*. [15] demonstrated that intact cells and cellular fragments of the saxitoxin producing dinoflagellate *Alexandrium tamarense* inhibited hatching success and larval survival of the scallop *Chlamys farreri. Heterosigma carterae* was found to suppress egg hatching rates when fed to the copepod *Acartia tonsa* [16] and severe reductions in copepod egg production rates were recorded during cyanobacterial blooms in the Baltic Sea [17]. Turner *et al.* [18] proposed that ingested phycotoxins may adversely affect successive generations of herbivores. Domoic acid is the most recognised and established diatom biotoxin and has been investigated in relation to anti-herbivory functionality [19–21]. However, from the perspective of reproductive impacts, it is the less-well-known oxylipins that are of prime interest. Oxylipins do not induce the same acute toxicity syndromes as the more commonly recognised algal biotoxins, and this has prompted their impacts on grazers to be referred to as ‘insidious’ [4]. They are however broadly cytotoxic with potential molecular targets associated with the cytoskeleton, calcium signalling and cell death pathways. These are unusual biological activities for microalgal-derived compounds which have also prompted some investigators to question whether or not the oxylipins are indeed toxins in the conventional sense. Toxins or not, it is precisely their bioactivity that has prompted current interest in exploring the scope and potential of these molecules for biotechnological and pharmacological development.

**Figure 1:**
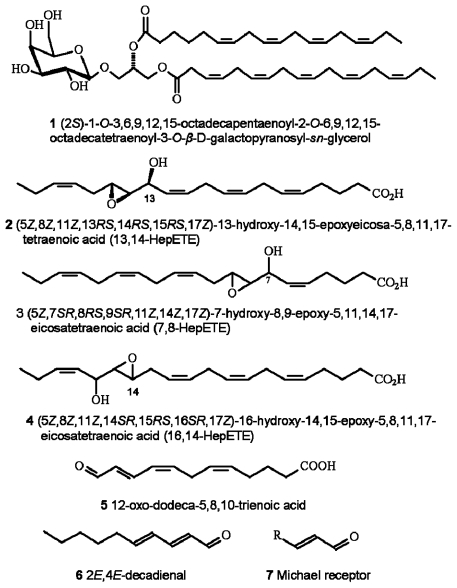
Chemical structures of selected diatom-derived molecules with bioactivities associated with cellular apoptosis and disruption of invertebrate reproductive processes. Structures redrawn from [27,28,30,88].

Oxylipin production in diatoms is wound-activated, meaning that synthesis is only initiated subsequent to loss of membrane integrity, typically as would occur during predation. Production and release of oxylipins, including polyunsaturated aldehydes (PUA) (which are the most intensively researched of the diatom-oxylipin family), hydroxyl-, keto-, and epoxyhydroxy fatty acid derivatives (see Figure 1) is extremely rapid. Production is by enzymatic oxidation of precursor polyunsaturated fatty acids and phospho- and galactolipids [22–26]. There is also evidence that some of the precursor compounds such as the galactolipid (2*S*)-1-*O*-3,6,9,12,15-octadecapentaenoyl-2-*O*-6,9,12,15-octa-decatetraenoyl-3-*O*-*β*-d-galactopyranosyl-*sn*-glycerol (**1**), may also be directly involved in cytotoxic reactions [27]. In addition, a further group of bioactive-molecules has recently been described [28] including a range of highly reactive oxygen species (ROS) and fatty acid hydroperoxides. They are formed from lipoxygenase-mediated oxidation of polyunsaturated fatty acids **2–4** including production from some non-PUA-producers. The resultant cocktail of ROS and oxylipins are highly damaging to invertebrate reproduction.

Of the oxylipins thus far described it is the PUA that have been the most comprehensively studied. This is partly due to PUA being the first group described [4], but also most PUA are commercially available, inexpensive and sufficiently stable to allow for a range of laboratory bioassays to be conducted. In contrast, many of the compounds described by Fontana *et al*. [28] and many of the intermediary compounds observed during PUA-synthesis are extremely unstable, require direct isolation from the algal source material and by default are neither readily available nor particularly amenable for biological testing. Of the PUA the C_10_ decadienal has received most attention and has been treated almost as a model PUA, despite the fact that this compound would appear to be produced in rather smaller quantities by diatoms compared with other PUA. Whereas PUA-based investigations dominate the literature and will be the main focus of this article, they may not necessarily represent the primary chemical strategy employed by diatoms; for instance, in a survey of 51 diatom species only 38% were identified as PUA-producers [29]. In addition, the benthic diatom *Phaeodactylum tricornutum* has been identified as a producer of the oxo-acids 12-oxo-(5*Z*,8*Z*,10*E*)-dodecatrienoic acid (12-ODTE) (**5**) and 9-oxo-(5*Z*,7*E*)-nonadienoic acid (9-ONDE), which also inhibit invertebrate embryonic cleavage [30]. The mechanisms described by Fontana *et al*. [28] may also help to explain a number of observed reproductive failures in non-PUA-producing diatom species. Assumptions cannot be made in relation to the relative importance or prevalence of one oxylipin system versus another due to limited data particularly for ROS production. As such, the combined contribution of PUA, ROS and other bioactive oxylipin production to diatom chemical defence is unknown.

Miralto *et al.* [4] isolated three C_10_ PUA (2*E*,4*E*-decadienal **6**; 2*E*,4*E*,7*Z*-decatrienal and 2*E*,4*Z*,7*Z*-decatrienal) from the bloom forming diatoms *Thalassiosira rotula*, *Skeletonema costatum* (now reclassified as *S. marinoi,* [31]) and *Pseudonitzschia delicatissima*. Subsequently an expanded range of PUA were identified including 2*E*,4*E*-heptadienal; 2*E*,4*Z-*octadienal; 2*E*,4*E*-octadienal; 2*E,*4*E*-2,4,7-octatrienal and 2*E*,4*Z*-decadienal [22,23,30,32–34]. Production of low-molecular-weight aldehydes by microalgae has been reported before [35–38], however it was not until the Miralto *et al*. [4] study that interest in these compounds began to blossom. It has now become apparent that the 2*E*,4*E* isomer is detected as an artifact, and that the diatoms produce the 2*E*,4*Z* isomer. The double bond geometry has no influence on PUA-bioactivity [39], rather the activity is primarily due to the α,β,γ,δ-unsaturated aldehyde reactive Michael acceptor element **7**, *i.e.,* the conjugation of a carbon–carbon double bond to the aldehyde functional group. The Michael acceptor can form covalent adducts with nucleophiles such as amines and is thus associated with toxicity [40,41]. The side chain polarity is also important for bioactivity with longer-chain PUA being more toxic. Prior to identification from diatoms, decadienal was recognised as possessing a range of biological activities including anti-mitotic and pro-apoptotic properties [42,43]. PUA are also recognised for their antibacterial and allelopathic activity [44–47], are genotoxic [48–50], and have been reported as cytotoxic to mammalian cells forming endogenous DNA adducts [51,52]. PUA are also associated with carbon tetrachloride-induced liver cirrhosis in rats [53]. Decadienal also inhibits tumour-necrosis factor-alpha mRNA, so modifying cytokine secretion by macrophages [54] and possibly contributing towards atherosclerosis [55,56] and carcinoma [57,58].

Aside from toxic and disease related properties, PUA also have important biological and ecological functions. Examples include forming a component of the defensive glandular secretion of *Hoplocampa* sawfly larvae acting as an allomone against epigaeic predators such as ants [59]. Both (*E,E*) and (*E,Z*) isomers of decadienal have been identified from the marine insect *Trochopus plumbeus* and are speculated to function as a pheromone [60]. Other aldehydes such as 10*E*,12*Z*-hexadecadienal and 10*E*,12*E*-hexadecadienal are important sex pheromones of the pyralid lepidopteran, *Notarcha derogata* [61]. There is also some evidence that decadienal can function as an attractant in a bait-type insecticide against adults of the western corn rootworm (Coleoptera: Chrysomelidae) [62]. Citral, a methylated form of octadienal (3,7-dimethyl-2,6-octadienal), has been shown to block limb development in chick embryos [63] and is functional as an alarm pheromone in mites [64]. Heptadienal may have a pheromonal function in the swallowtail butterfly, *Papilio machaon* [65]. 12-ODTE has been proposed as a precursor of an algal sex pheromone [66]; is cytotoxic [67]; and induces a rapid increase in cytoplasmic free calcium in human blood neutrophils [68].

Oxyplins, and PUA in particular, have important biological and biochemical properties. PUA disrupt a number of salient stages in reproductive and developmental processes including; gametogenesis, gamete functionality, fertilization, embryonic mitosis, and larval fitness and competence [5,69–75]. It has been suggested that PUA-production, via reproductive interference, could facilitate bottom-up control of grazer populations by limiting recruitment [4,5,76]. The use of aldehydes as defensive compounds in terrestrial systems and by macroalgae is well known [77–82]. These defensive aldehydes are often biologically-active in a variety of different ways *e.g.,* antimicrobial; embryotoxic; sperm motility inhibitors; larval-toxic; grazer-toxic, allelochemicals and feeding deterrents [81]. Latterly, there has been considerable attention devoted to documenting the effects of diatom-grazing on the population dynamics of copepods [5,12,26,83–85] and also on elucidating the various biosynthetic pathways of oxylipin formation [24,25,28,32,86]. Both lines of enquiry have also been the subject of recent and pertinent reviews [76,87–89], however, despite the apparent criticality of reproductive interference there has been comparatively little effort directed towards elucidating the modes of action of these chemicals at the physiological, cellular and molecular levels.

The majority of work addressing copepod secondary production has applied egg production as the metric of choice [90,91]. If taken in isolation egg production would have resulted in negative diatom effects being overlooked, as in general egg production is not consistently affected, although there are exceptions in the literature. An influential study by Ban *et al*. [92] presents strong evidence for the deleterious effects of diatoms on copepod reproduction including fecundity (eggs/female/day). The authors identified 4 copepod reproductive response categories in relation to diatoms: 1) reduced fecundity and hatching success; 2) reduced hatching success; 3) reduced fecundity; or 4) no negative effect. This paper will attempt to summarize current understanding of the impacts of oxylipins on invertebrate reproduction and development, but will not consider egg production further given the lack of clear and consistent relationships. This work will focus specifically on impacts on gametogenesis, gamete functionality, embryogenesis and larval fitness. The majority of studies cited are copepod orientated, however significant progress has been made using alternative model organisms such as echinoderms, ascidians and polychaetes. Evidence collected from all species will be used to explore common patterns of oxylipin bioactivity.

## 2. Gametogenic Effects

There have been a number of documented examples of direct impacts of oxylipin exposure and/or diatom ingestion on gametogenesis; primarily oogenesis and oocyte maturation. The relative paucity of literature in this area is principally due to the difficulties of studying gametogenic processes in copepods; however application of confocal laser scanning microscopy coupled with specific fluorescent probes has proven an effective and instructive tool for investigation [75,93,94]. The oogenic- and endocrine-cycles in copepods are particularly understudied in comparison to higher crustaceans and insects. Gonadal development stages have been described for a limited number of copepods including *Calanus* and *Pseudocalanus* species [95–97]. A degenerative response of ovarian tissue as a direct response to diet has recently been documented by Poulet *et al.* [98]. The study of gonad and gamete development is particularly important when considering the potential effects of dietary-sourced developmental inhibitors as contact with the gonads will most likely be the first association between inhibitory compounds and the reproductive system.

### 2.1. Oogenesis and Oocyte Maturation

Information concerning oxylipin-effects on oogenesis and oocyte maturation is scant. Ultrastructural examination of the ovaries of the copepod *Temora stylifera* fed diatoms known to affect hatching indicated that a diatom-diet does not affect gonadogenesis when compared to wild fed controls [99]. However, Buttino *et al*. [75] deployed liposomes to deliver known quantities of decadienal directly into the gut of *Calanus helgolandicus* and *T. stylifera* females and observed that decadienal initiated apoptosis in both somatic and gonadal tissues (also see Wichard *et al.* [100]). This provides compelling evidence that PUA-ingestion can have a discernable effect on ovarian architecture which may in turn affect either/or fecundity or egg viability. There was also a clear effect on adult survivorship with mortality linked with apoptotic events in the somatic tissues. Interestingly, Poulet *et al.* [98] described two separate oogenic syndromes in *C. helgolandicus* as a result of diatom-feeding, although in this case the indicative species were not those associated with PUA-production. Syndrome one was characterised by reduced egg production whereas egg hatching was unaffected. In syndrome two however, hatching success and teratogenesis were symptomatic. The described syndromes are in agreement with the responses described by Ban *et al.* [92]. Reduced egg production was coincident with apoptotic degradation of the oocyte at development stage 3 bringing about developmental arrest at stage 4. The capacity of an oocyte to fertilize is critical. In calanoid copepods fertilization occurs at stage 4 as the oocyte passes the spermatheca [97,101,102]. Toxic effects during oogenesis or at oocyte maturation may have serious implications for fertilization efficiency.

**Figure 2:**
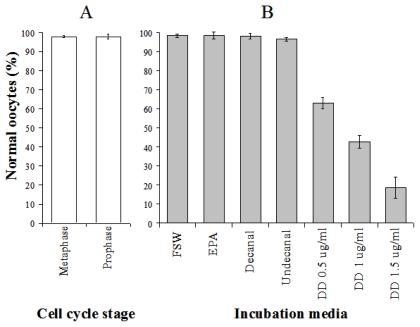
The percentage of morphologically normal oocytes of *Asterias rubens* **(**a) following *in vitro* exposure to 1.5 μg mL^−1^ decadienal at either the prophase or metaphase cell cycle stages and (b) exposed during meiotic-reinitiation to either filtered seawater (FSW), *cis*-5,8,11,14,17-eicosapentaenoic acid (EPA), the saturated-aldehydes decanal and undecanal or the PUA decadienal (DD) at concentrations of 0.5, 1 or 1.5 μg mL^−1^. Mean (±S.D.) of 3 replicates. Details of experimental design can be found in Caldwell [103].

**Figure 3:**
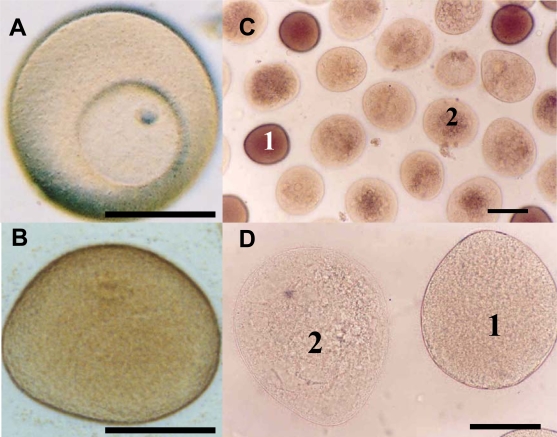
*Asterias rubens* oocytes exposed to decadienal at a concentration of 1.5 μg mL^−1^ (a) control prophase oocyte displaying intact germinal vesicle, (b) control metaphase oocyte having undergone germinal vesicle breakdown, (c&d) oocytes exposed to decadienal at a concentration of 1.5 μg mL^−1^. 1 = morphologically normal oocyte, 2 = necrotic oocyte. Scale bar = 100 μm. Details of experimental design can be found in Caldwell [103].

Caldwell [103] investigated the effects of *in vitro* exposure to decadienal during oocyte meiotic-reinitiation (prophase-metaphase transition) in the common starfish, *Asterias rubens*. During oogenesis, starfish oocytes arrest at the end of the first prophase of meiosis. Prophase-arrest is maintained until the oocyte is ready to be spawned. For successful maturation and development, the oocyte must exit prophase-arrest and complete meiosis. The maturational stimulus can be in a variety of forms depending upon the species in question including; fertilization, hormonal action, limited proteolysis and release from an inhibitory stage. *Asterias rubens* induces oocyte maturation by the action of the follicular-derived hormone 1-methyladenine [104] allowing for simple maturation *in vitro*. Oocytes exposed to decadienal during either prophase or metaphase were unaffected (Figure 2A), however, a cytotoxic response was induced when prophase oocytes were stimulated to undergo maturation (*i.e.*, reinitiation of meiosis) at concentrations between 0.5 and 1.5 μg mL^−1^. At a concentration of only 0.5 μg mL^−1^ between 30 and 40% of oocytes had undergone cell death. At the maximum decadienal concentration assayed (1.5 μg mL^−1^) between 80 and 90% of oocytes were rendered unviable (Figure 2B). Figure 3 shows oocytes that were exposed to 1.5 μg mL^−1^ decadienal during maturation. The cytoplasm tended to become lighter in colour and more globular towards the centre. Also the overall oocyte diameter increased. Similar observations were reported by Poulet *et al*. [98]. Using morphological criteria, the oocytes were determined to have undergone necrotic cell death as opposed to apoptosis; however additional diagnostic tests were not performed to confirm this. A typical necrotic response involves swelling and subsequent rupture of the nucleus, chromatin condensation, rupture of the nuclear envelope and expulsion of its contents into the cytoplasm, with merely a faint impression of the organelle remaining [105]. The saturated aldehydes decanal and undecanal or the fatty acid eicosapentaenoic acid had no discernable effect. When maturation was initiated in the presence of decadienal, the oocytes underwent a severe cellular disruptive event. Decadienal therefore is cytotoxic during the prophase/metaphase transition and may have an important role in determining oocyte viability in diatom-feeding invertebrates.

Cell death generally occurs via one of two possible pathways; apoptosis and necrosis. Apoptosis, also referred to as programmed cell death, is considered ‘physiological’ cell death and generally occurs in an organism to maintain its function. Necrosis is linked to pathological inflammatory conditions following exposure to extreme physical, biological or chemical conditions. Cell death in adults, gonads, early embryonic stages and larvae in response to decadienal exposure has been described previously [5,69,75,98], however in these cases, cell death was determined to have followed an apoptotic pathway. A combination of both apoptosis and necrosis was described by Poulet *et al*. [106] in copepod nauplii spawned from mothers feeding on a PUA-producing diatom strain. It has now been demonstrated that sea urchin embryos exposed to decadienal during early ontogeny are unable to activate the G_2_-M phase promoting-complex cyclin B-Cdk1 despite an accumulation of cyclin B [107]. The G_2_-M phase promoting-complex is also essential for oocyte maturation in several marine invertebrates [108]. It is therefore likely that decadienal is acting against the maturation-promoting-complex in oocytes of *A. rubens* and probably copepods, thereby initiating a terminal cellular response.

Exposure to decadienal is known to inhibit the plasma membrane voltage-gated calcium current in ascidian embryos [70]. Calcium, released intracellularly in starfish plays an important role in meiotic-reinitiation. Maturation can be blocked by calcium inhibitors or induced by calcium-ionophores, iontophoresis, or increased calcium concentration in the external medium [109]. Calcium overload or perturbation of intracellular-calcium-compartmentalization is known to initiate cytotoxicity, resulting in cell death by either apoptotic or necrotic pathways [110]. It is possible that interference with calcium signal transduction may contribute towards the documented cell death response hence rendering the oocytes nonviable. Cytotoxicity at oocyte maturation may represent an additional and hitherto under considered mechanism whereby PUA may limit grazer population growth.

To exert an effect on oogenesis and oocyte maturation the oxylipins must come into direct contact with the gonadal tissues. Poulet *et al*. [111] suggest that inhibitory compounds are accumulated in oocytes following diatom-feeding. Copepods are known to accumulate dinoflagellate toxins and domoic acid within their tissues to concentrations lethal to their predators but that do not affect copepod survivorship [20,112]. The copepod gut epithelium is in close contact with the ovary, so a diffusion mechanism would appear plausible. Circumstantial evidence for this comes from the time lag between initiation of a diatom-diet and the production of non-viable eggs. There appears to be a lag phase of 24 to 72 h during which time viable eggs are produced, followed by progressive reduction in oocyte viability. Additionally, Laabir *et al*. [113] demonstrated that by reducing diatom cell densities from 10^5^ to 10^4^ cells mL^−1^, the time required to reach total egg inhibition was increased from 7 to 12 days.

To accumulate within, or be associated with the ovaries the oxylipins must by necessity traverse a number of lipid membranes. The transportation of non-polar hydrocarbons across membranes is known to occur, for example, the perception of pheromone signals in macroalgae. The degree of incorporation is governed by the characteristic partition coefficient between water and the lipid membranes [114,115]. Importantly, trans-lipid transportation of decadienal has in fact been demonstrated by Trombetta *et al*. [45] using liposomes and latterly by Buttino *et al*. [75]. Therefore, gonadal exposure is a viable explanation for observed laboratory feeding trials. However, it is unlikely that the oxylipins are accumulating in the tissues due to the high reactivity of the molecules which will readily attack the amine groups of amino acids, proteins and enzymes to form imines or Schiff bases [44,116]. It is more feasible that low level molecular and cellular damage is occurring such as DNA damage, and where the rate of damage exceeds the capacity of the adult to repair the damage, reproductive effects may be observed. Such damage is particularly relevant for oocyte maturation. Inhibitors of protein kinases are known to affect oocyte maturation in a number of marine organisms including starfish and molluscs [117]. If this damage is retained in the early embryo, particularly given the lack of cellular checkpoints until the mid-blastula transition, subsequent transcription errors may induce further developmental problems including induced apoptosis (see embryogenesis section for further discussion of DNA damage and cell cycle checkpoints).

### 2.2. Spermatogenesis and Sperm Motility

The study of diatom-derived developmental inhibitors has focused almost exclusively on maternal aspects, a trend that may best be illustrated by the following quote, “Components of the food that are potentially toxic to egg hatching are, by necessity, mediated by the female...” [8]. Maternal sources will undoubtedly be of primary importance, however, impacts attributable to the paternal line must also be considered. Whereas maternal contributions to overall copepod reproductive success have been heavily researched the male contribution has received scant attention. This is likely due to the relative ease of manipulating and incubating oocytes, particularly in broadcast spawning calanoids, compared with the rather more challenging nature of working with spermatophores. Another factor working against sperm research is the immotile nature of crustacean sperm (with the possible exception of some cirripede species [A.S. Clare, personal communication 2009]). For species spawning motile sperm experimentation and monitoring are comparatively straightforward, for example, the field of invertebrate sperm ecotoxicology has benefitted greatly from the application of computer-assisted sperm analysis (*e.g.*, [118,119] and see Figure 5). The need for adequate recognition of the male condition in relation to copepod reproduction has been acknowledged by Titelman *et al*. [120]. Indeed, it is widely recognised that there is a clear knowledge gap in copepod reproductive biology in relation to spermatology.

**Figure 4:**
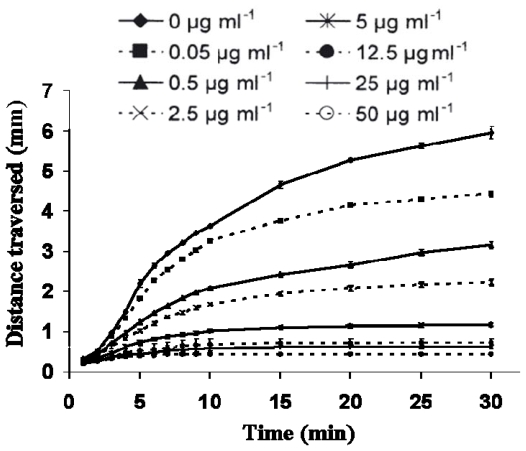
The effect of increasing concentration of decadienal on *Asterias rubens* sperm-front migration velocities. Mean value of three replicate experiments for each concentration ± S.D. Details of the experimental design can be found in Caldwell [103] and Caldwell *et al*. [72].

**Figure 5:**
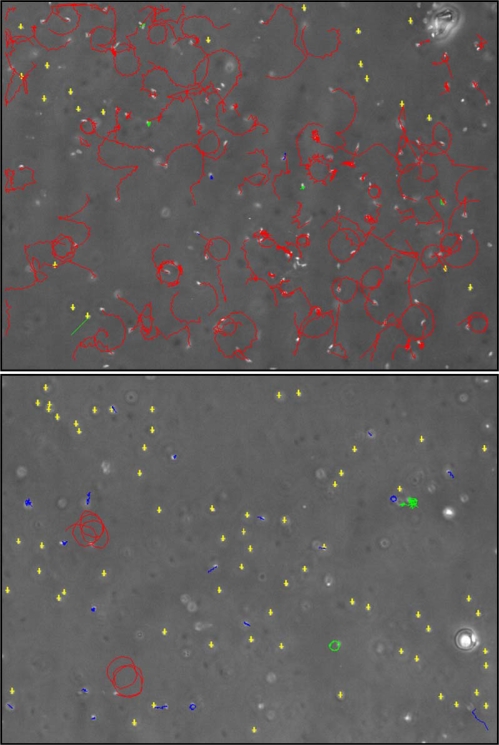
Screen grabs of *Nereis virens* sperm subjected to computer-assisted analysis under exposure to (a) filtered seawater control and (b) 5 μM decadienal [184].

In relation to possible inhibitory dietary-effects on copepod sperm, laboratory feeding trials have tended not to rear males on diatom-diets but rather used wild males that had been feeding on varied natural phytoplankton. However, it has previously been demonstrated that male copepods fed particular dinoflagellate diets had a reduced fertilization capacity [7,121] and reduced spermatophore production, however ultrastructure examination did not reveal any obvious anomalies. Ianora *et al*. [121] did suggest that sperm quality may have been affected during spermatogenesis given the strategy of *Calanus helgolandicus* females to store mature sperm. These studies highlight the contribution of sperm quality in determining reproductive success in copepods. Whereas neither study addressed diatom-oxylipin-effects *per se*; there is compelling evidence of pronounced effects of PUA on sperm motility ([72,103,184]; and Figures 4–5). Decadienal inhibits motility in a clear dose- and time-dependent manner for a range of broadcast spawning marine invertebrates, but importantly does not result in sperm death. The inhibitory response is extremely rapid with sperm curvilinear velocity reduced by almost 75% within three minutes of exposure to 0.1 μM decadienal [Caldwell *et al*. in prep]. What is particularly unusual is that the sperm retain the capacity to fertilize if exposed to manual mixing (*i.e.,* a proxy for cellular motility). Figure 5 provides a good visual indicator of a typical three minute motility-inhibition response, in this case using 5 μM decadienal. Whereas it can be seen that not all sperm are immobilised, the vast majority are. All cells remained viable as the sperm heads were observed to twitch, however flagellum function was lost. This loss of motile function without any obvious structural anomalies corroborates observations made on copepod sperm. This example of an algal compound affecting sperm motility is not unique, however it is rare. Wicklund [122] found that crude extracts from fucoid macroalgae blocked sperm/egg interactions but did not inhibit sperm motility; on the contrary, the period of motility was prolonged. Other macroalgal metabolites, including aldehydes such as udoteal and halimedatrial, are known to inhibit sperm motility [81]. Extracts of *Phaeocystis pouchetti* have also been demonstrated to inhibit sperm motility [123] however, these have since been determined as containing decadienal [124].

As mentioned previously, PUA have been shown to inhibit voltage-gated calcium currents [70]. Calcium ions play an essential role in the normal functioning of marine invertebrate sperm, greatly influencing the pattern and shape of flagellar bending and thereby exerting a control over swimming behaviour [125,126]. Disruption of calcium signal-transduction will affect sperm function, for example, in sea urchin sperm, elevated calcium increases flagellar beat asymmetry and reversibly blocks beating at 0.1–0.2 mM [125,127,128]. In addition to calcium interference PUA also interfere with the stability of microtubulin and actin filaments [70,107]. Tubulin dynamics are also critical for sperm motility [129]. Any chemical agent which alters microtubule stability, colchicine for instance [130], will severely hamper sperm functionality.

Sperm motility is an essential prerequisite for fertilization in broadcast spawning animals such as echinoderms, but not necessarily for crustaceans. Crustacean sperm is commonly aflagellate and therefore non-motile. This fundamental distinction between species employing motile versus non-motile sperm makes extrapolating results from echinoderms to copepods rather difficult. An important question to pose is whether a compound that inhibits sperm flagellum function would have any impact on sperm function in aflagellate sperm? As demonstrated by Ianora *et al.* [121] there is a connection between diet, spermatophore production and sperm quality. This may have particular significance for species that mate only once. Titelman *et al*. [120] provide some very interesting discussion as to whether male copepods are the limiting sex; including the energetic cost of spermatophore production and whether males provide nutrient and/or hormonal supplements to the female. Perhaps we can progress towards adding dietary-induced male sterility to the discussion? Could this also be a driver for the evolution of multiple matings, female selection and sperm competition in copepods? Given the clear research bias toward copepod females a change in mindset is needed if the nature of the male condition is to be understood.

## 3. Fertilization

Fertilization is the union of two sexually-differentiated haploid gametes to form a diploid zygote. For sexually reproducing organisms this is a critical stage in the reproductive process. Fertilization is a complex multi-step process, which from the perspective of chemical intervention, presents many targeting opportunities.

Fertilization strategies may be broadly categorised as internal and external. For animals exhibiting internal fertilization sperm are transferred directly from male to female by a variety of mechanisms, with subsequent fertilization occurring inside the female body. Assuming gamete competency, any chemically-induced disruption (oxylipin or otherwise), would therefore largely be attributable to agents present either within the body or sequestered within the gametes. There would be limited opportunity for a direct, point-of-contact chemical intervention. In contrast, external fertilizers (*e.g.,* broadcast spawners) release their gametes freely into the environment with fertilization occurring independent of further parental influence. The germ cells of external spawners would therefore be significantly more vulnerable to fertilization failure as a result of point-of-contact chemical exposure.

References to the effects of algal toxins on fertilization biology are relatively few. Examples include; toxins produced by the haptophyte *Chrysochromulina polylepis* that inhibit fertilization in ascidians and mussels [131] and the ciguatera poison, maitotoxin, is known to inhibit sea urchin fertilization by oocyte exocytosis [132]. Diabolin, a 120K protein from the kelp, *Laminaria diabolica* activates elevation of the fertilization membrane in unfertilized sea urchin eggs [133]. As discussed previously, Ianora *et al*. [121] and Laabir *et al*. [99] describe adverse effects on fertilization success due to poor sperm quality in the copepod *Temora stylifera* reared on certain dinoflagellate diets. Diatom-oxylipins are the most comprehensively researched in relation to fertilization effects. While feeding on the diatom *Phaeodactylum tricornutum*, eggs of *Calanus finmarchicus* were spawned which had not completed pronuclear fusion [134]. Buttino *et al*. [135] also observed failed pronuclear fusion in sea urchin eggs exposed to diatom extracts. Hansen *et al*. [123] observed reduced fertilization success in sea urchins exposed to extracts of *Phaeocystis pouchetii -* the active component subsequently recognised as decadienal. Caldwell *et al.* [71] demonstrated that fertilization success was reduced by exposure to decadienal, with the impact reduced by sequential washings. Oocytes incubated with decadienal over set times were not greatly affected when fertilized with untreated sperm. The fact that oocytes treated with decadienal and subsequently washed retained a high fertilization rate suggests that decadienal does not strongly associate with the oocyte surface-membrane where it may have affected sperm/egg binding. Sperm incorporation and the formation of the fertilization cone proceeded as normal in oocytes treated in this way, in contrast with fertilizations in the presence of decadienal above a concentration of 1 μg mL^−1^. A similar observation was made by Tosti *et al.* [70] whereby decadienal interfered with actin microfilament reorganisation. The cytoskeleton appears to be a crucial target for PUA. The implications of this in relation to fertilization biology are profound. A coordinated sequence of motility events mediated by both microtubules and microfilaments are required for successful fertilization. Microtubules are the functional cytoskeletal component during pronuclear migrations and syngamy, whereas microfilaments mediate extrusion of the sperm acrosomal process, the formation of the fertilization cone and the block to polyspermy [136,137]. Schatten and Schatten [137] demonstrated that sperm incorporation was possible in oocytes treated pre-insemination with microtubule-inhibitors, whereas microfilament-inhibitors prevented incorporation.

Tosti *et al.* [70] made significant progress in explaining the potency of decadienal and similar PUA against early fertilization processes. Decadienal and decatrienal were observed to inhibit the fertilization-current generated at the point of sperm-egg interaction. Concomitant inhibition of plasma membrane voltage-gated calcium currents was also noted. Decadienal was identified as a specific fertilization-channel-inhibitor as it did not perturb either gap-junctional communication or plasma membrane steady state conductance. Decadienal appears to be the first documented fertilization-channel blocker in marine invertebrates [70].

The extent of *in situ* fertilization impacts as a result of diatom-oxylipins is unknown and there is currently insufficient data to even begin to develop projections. What is clear is that diatom-oxylipins have distinct biochemical properties which impact fertilization processes either by impairing gamete functionality and/or -competency or by disrupting salient stages during the early fertilization process. Environmental exposure to oxylipins may be dietary or by direct encounter with spawned gametes, particularly if spawning coincides with a diatom or *Phaeocystis* bloom (see [71] for further discussion). The enzymes involved in PUA-synthesis remain active for a considerable period of time after cell-wounding (see [23]), resulting in a ‘cloud’ of PUA associated with the dispersed algal cytoplasm. This may potentially exert a significant environmental check on fertilization success.

## 4. Embryogenesis

Development may be considered at a number of levels, ranging from the rapid mitotic cleavages of embryogenesis, the metamorphosis between larval stages, the formation of an adult body plan, the development to reproductive maturity and the continuous repair of somatic tissue during life. The life-cycle phases which are most susceptible to stress are the earliest (embryo and larvae) and the latest (senescent).

Arrested embryogenesis has frequently been reported in response to both diatom-feeding, exposure to diatom extracts and direct exposure to diatom-derived oxylipins. The initial report was in 1994 by Poulet [111] and co-workers who observed aborted development in embryos of the copepod *Calanus helgolandicus*. The point of arrest was variable but tended to be between just prior to pronuclear fusion or at various subsequent mitotic cleavages. The authors describe a number of cellular aberrations including globular cytoplasm and dispersed chromatin. Poulet *et al*. [134] describe cytological disruption in embryonic development during incubation in diatom extracts. Developmental abnormalities arose from an inability of the embryo to synchronise nuclear division with intercellular membrane formation during mitosis resulting in either developmental-arrest at the zygote stage or continued but abnormal development. Similarly, Buttino *et al*. [135] documented errors during embryogenesis resulting in hatching inhibition in sea urchins and ascidians incubated with extracts of the diatom *Thalassiosira rotula*. Extracts were observed to block tubulin organisation, promote microtubule depolymerisation and prevent chromatin condensation. Microtubule depolymerisation was observed from pronuclear fusion to telophase resulting in blockage of cell division. Nuclear fragmentation without cytokinesis was also observed. At high cell densities (5 × 10^6^ and 10^7^ cells mL^−1^) the cell cycle was inhibited at the first mitotic division while at lower cell densities (2.5 and 1.25 × 10^6^ cells mL^−1^) the majority of embryos completed first cleavage with a number developing to the eight blastomere stage. The pathology described by Buttino *et al.* [135] for *Paracentrotus lividus* embryos was very similar to that described for *Calanus helgolandicus* by Poulet *et al.* [134] suggesting a homogeneous mode of action.

Romano *et al.* [69], using a suite of techniques, confirmed that embryotoxicity was linked to apoptosis, however differences in response were noted between copepod and sea urchin models. Copepods appeared to follow a caspase-independent path whereas a caspase-3-like protease was activated in sea urchins. Using *Calanus helgolandicus* and *Temora stylifera*, Buttino *et al.* [75] observed apoptosis in embryos spawned from mothers that had ingested decadienal encapsulated in liposomes.

Again, using a sea urchin model, Hansen *et al*. [107] characterised a range of mitotic cellular events that were disrupted due to PUA exposure, including; inhibition of pronuclear migration; lack of DNA replication; tubulin depolymerisation; and importantly the failure to activate the G_2_-M promoting-complex cyclin B-Cdk1. This final observation is potentially very important. M-phase promoting-factor (MPF) and cyclin-dependent kinases are essential for mitotic progression in the embryo. Interference with the synthesis or activity of either will have dire consequences, for example see Abraham *et al*. [117] who observed a range of effects associated with the inhibition of cyclin-dependent kinases in a range of eukaryotes. Interestingly, that study is, as far as I am aware, the only published work on the role of cyclins in copepod mitotic progression. It is unclear as yet whether PUA are acting directly on this complex or are affecting upstream cellular processes, perhaps one that is governed by cytoskeletal function which may have been compromised.

Cellular extracts from *Skeletonema costatum* have been shown to inhibit tumour cell proliferation through inhibition of normal cell division at G1 phase [138]. Both decadienal and decatrienal inhibited proliferation in Caco2 human colon adenocarcinoma cell lines [4]. The production of anti-mitotic compounds by plants is widely recognized, many of which have subsequently been exploited by the pharmaceutical industry, including some of the most valuable cancer treatments. Examples include: colchicine from the autumn crocus (*Colchicum autumnale*); podophyllotoxin from the lapacho tree (*Tabebuia* sp.); vinblastine and vincristine from the Madagascar periwinkle (*Catharanthus roseus*); and paclitaxel (Taxol®) from the Pacific yew (*Taxus brevofolia*). Colchicine interferes with the binding of tubulin-Guanosine triphosphate, which prevents microtubulin formation [139]. Colchicine is also an aneugen, a substance causing numerical chromosomal aberrations, frequently resulting in cell death [140]. It is therefore unsurprising to learn of potential anti-mitotic compounds from microalgal sources.

Interference with microtubule function in the activating oocyte and early embryo can affect localized RNA transcription which may in turn affect polarity and patterning [141]. Laabir *et al*. [99] suggest that cytoskeletal disruption may interfere with protein and amino acid biosynthesis, therefore affecting organogenesis in copepods. The authors noted sudden sharp variation in several amino acids coinciding with abortion of early embryos. Damage to DNA and RNA is potentially disastrous for rapidly dividing embryos, many of which appear to lack the repair checkpoints of somatic cells until the mid-blastula transition [142]. As discussed in relation to sperm motility and fertilization, the cytoskeleton is intrinsic to virtually all cellular functions, including chromosome separation, intracellular vesicle transport and determination of cell shape. The thiol-groups contained within tubulin are important for its function and are suspected targets for PUA-reactivity [143]. A compound that targets and inhibits the dynamic functioning of the cytoskeleton can essentially bring all cellular processes to a halt. To use a rather crude metaphor, it would be like trying to play Handel’s Concerto in B flat on a harp that has had all the strings cut. Therefore, the production of compounds that target your predator’s ability to function on a cellular level can be a highly effective anti-grazing strategy.

Caldwell *et al.* [71] noted that exposure to decadienal at sublethal concentrations slowed the rate of embryonic development. In effect, exposure prolonged the duration of the embryonic and larval phases. Egg viability is a crucial concept in relation to reproductive and population fitness. This has been demonstrated in fish [144–146] where it was observed that poor egg quality reduced the survival rate of hatched larvae by extending the length of the larval phase and hence the succeeding generation. Frangópulos *et al*. [147] reared *Acartia clausi* on combinations of toxic and non-toxic dinoflagellates, and noted that nauplii hatched from toxin-fed mothers required more time to moult to the copepodite stages. Sellem *et al.* [148] described abnormal development in embryos and larvae of the sea urchin *Paracentrotus lividus* incubated with the fatty acid octadecapentaenoic acid derived from the dinoflagellate *Gymnodinium* cf. *mikimotoi*. Development rate was retarded during the differentiation stage. The time delay was compensated for during the proliferating stages allowing the larvae to synchronously metamorphose to the pleuteus stage. Similar observations were made by Gentien *et al*. [149] on *Mytilus* sp. embryos incubated with *Gymnodinium* cultures.

**Figure 6:**
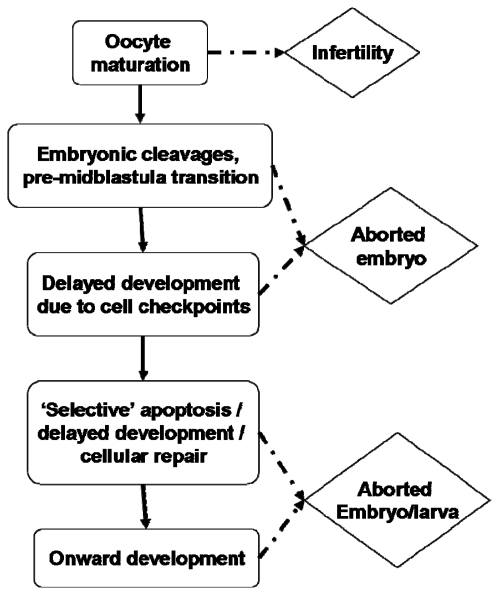
Stagegate model for an embryo affected by pro-apoptotic diatom compounds during development. The initial checkpoint is whether the cell is competent to undergo meiotic-reinitiation. The consequence of failure is oocyte apoptosis, which for many species would then trigger reabsorption of the oocyte nutrients by the mother. Genetic surveillance activated at the midblastula transition may trigger abortion or arrested development. Surviving larvae may then undertake a programme of ‘selective’ apoptosis to eliminate non-viable cells - the extent of such will determine whether the larva is competent for further development. See [5,98,135,142] for examples of each checkpoint.

Can these rate delays be rationalised? With regard to what is known of the effects of PUA on embryogenesis, perhaps consideration of cell cycle checkpoints may prove fruitful. It is known that decadienal can induce significant cellular abnormalities such as aneuploidy [150] and can inhibit DNA synthesis and replication [107]. Cellular mechanisms have evolved to prevent further cell division until any genetic damage has been repaired [151,152]. Ordinarily, apoptotic pathways would also be activated to remove non-viable cells or those beyond repair [153]. These checkpoints can have the effect of delaying development until the cell or embryo is competent to continue. However, it is generally regarded that checkpoints do not begin to function until the mid-blastula transition in rapidly dividing embryos [154]. Earlier mitotic divisions express an abridged cell cycle with highly compressed G_1_ and G_2_ phases, thereby allowing continuous cycles between S-phase (DNA replication) and M-phase (nuclear division). DNA repair would normally occur during the G phases, therefore cellular surveillance would not operate until numerous cycles of DNA replication and mitotic cleavage had occurred, with or without genetic damage. There is some suggestion that this generalisation may not hold entirely true for sea urchins [155,156], however most sources are in support of the critical stage of the mid-blastula transition [157,158] which leaves a considerable portion of early embryogenesis without an apparent mechanism for genetic surveillance. Le Bouffant *et al*. [156] suggest that the extent of DNA damage is an important factor in whether cell cycle delay or arrest is triggered. Relatively minor damage will cause a delay in cycle progression thereby allowing DNA repair to occur; more extensive damage initiates cell cycle arrest leading to an apoptotic response. Such observations would appear to hold true for PUA-toxicity; higher exposure is pro-apoptotic whereas lower exposure results in delayed cell cycle progression. Figure 6 illustrates what may be regarded as a series of gateway checkpoints through which the oocyte/embryo must pass during development. It would appear that this is not an either/or process in PUA-exposed embryos; severe exposure during early embryogenesis does result in developmental arrest and abortion, low-level exposure may allow development to progress unaffected with perhaps only minor rate delays. However, a potentially intermediary response with apoptosis restricted to badly damaged cells, the extent of which may or may not affect subsequent larval development and fitness, may explain teratogenic observations. An argument may therefore be made for potentially non-lethal, ‘selective’ or sacrificial apoptosis induced by environmental stressors. For this to be a realistic option, the larva must have cells or tissues that can be sacrificed. It would therefore seem logical that as the larva grows in size the potential to survive apoptotic events increases; in other words the larva has an increased capacity for non-mortal tissue sacrifice. Such a response may be important for the evolution of counter defences assuming the animal can then develop to reproductive maturity. However, as will been seen from the following section, surviving cell checkpoint apoptosis in the short term does not necessarily equate to survival in the longer term.

## 5. Larval Fitness

Exposure to harmful environmental agents during embryogenesis and larval development can affect subsequent development, growth and organism fitness. The degree of exposure to harmful compounds can be broadly classified as acute (rapidly lethal) or chronic (sublethal). In relation to environmental exposure, chronic exposure is the most frequently encountered situation. Exposure to sublethal levels of harmful agents can have significant effects on exposed animals including reduced immunological status, slower growth and abnormal development.

Evidently, the oxylipin impacted embryo must circumvent a number of critical developmental challenges if it is to survive to ‘larvalhood’. The embryo makes the transition to larva upon hatching; however, the hatching experience in itself represents a considerable hurdle. After the metabolic demands of embryogenesis the larva is then required to breach the confines of the oocyte membrane. Many species have evolved specialised appendages to aid hatching, such as the egg tooth in birds. Although the oocyte membrane in aquatic invertebrates will not provide the same degree of resistance as the calcium carbonate of a bird’s eggshell [159], hatching will nevertheless exert a metabolic toll. Hatching, therefore, represents a potential developmental bottleneck that may be further complicated by a weakened disposition due to toxin exposure. Indeed, Caldwell *et al.* [71] observed numerous fully formed larvae that were apparently morphologically normal but were unable to hatch.

Assuming successful hatching, the larva is then propelled into a world where it must face a new set of challenges; it must avoid predation; and, depending on species, be required to feed; undergo further development; potentially metamorphose; locate a suitable habitat; compete for space and resources; and ultimately recruit to the adult population. Considering that the larval phase can last from a few brief hours to a number of years depending on the species in question, it is fair to say that the traumas of embryogenesis are more than matched by the harshness of larval life. For larvae that embark upon this journey already carrying the burden of embryonic stress, the prospect of recruitment success is woefully slim.

**Figure 7:**
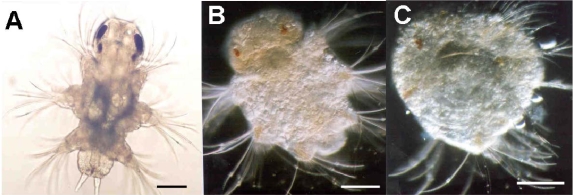
Induction of morphological abnormalities in 9 day old larvae of *Nereis virens* due to exposure to decadienal at concentrations of **(a)** 0, **(b)** 0.01 and **(c)** 0.05 μg mL^−1^ during embryogenesis. Details of experimental design can be found in Caldwell [103].

The recognition, documentation and certainly quantification of abnormal copepod larval forms are very much a contemporary development. Uye [160] found that nauplii hatched from diatom-fed females were generally deformed and expressed marked morphological asymmetry. Most deformed nauplii died after hatching, whereas those that survived displayed greatly impaired swimming behaviour and died shortly afterwards. Miralto *et al.* [161] recorded that 81.8% of spawned eggs of *Calanus simillimus* from the Straits of Magellan underwent abnormal development resulting in either abortion or production of asymmetrical larvae. Similar observations have been noted in a range of laboratory and field studies [28,85,98,113,134,162,163]. Poulet *et al.* [134] document the severity of swimming behaviour change as a direct consequence of abnormal asymmetric development whereby the nauplii could not maintain a linear heading but tended to turn in the direction opposite to the most developed swimming appendages. Such a change in locomotory capacity will impair feeding efficiency and escape behaviours [164,165]. Many of these studies report concomitant poor hatching success and larval survival with patterns of abnormal production often reversed if mothers were switched to non-diatom-diets. The study by Ianora *et al*. [5] highlights the complexity of this issue as, using *Calanus helgolandicus*, they observed arrested and abnormal development with high mortality rates in larvae spawned from diatom-fed mothers and for which the larvae were similarly diatom-fed. Larval survival was not improved even when the larvae were switched to a non-diatom-diet. Survival was improved when the maternal diet was changed even when the resultant larvae were diatom-reared, suggesting that teratogens consumed maternally are more critical that those consumed by the progeny. Impaired larvae were shown to be undergoing apoptotic events. Similarly, Caldwell *et al*. [71] described polychaete and echinoderm embryos that hatched following PUA-exposure which tended to display anatomical malformations. These included incomplete ciliary band formation in *Nereis virens* trochophore larvae, and stunted and asymmetrical larval arms in *Psammechinus miliaris* echinopluteus larvae. Developmental abnormalities were also carried into subsequent larval forms, for example, damage accrued during the trochophore stage was still traceable in the nechtochaete stage (see Figure 7). Phenotypic malformations resulting from environmentally realistic toxin exposures will impact upon larval fitness, subsequent recruitment success and population growth. Morphological abnormalities acquired during embryogenesis may be maintained by the individual throughout the remaining ontogenetic process. There is no evidence yet for this in copepods; however there does exist some circumstantial support. Williams and Wallace [166] identified a deformation rate of 1–8% of the fifth limbs in adult female *Clausocalanus* sp. but the origin of this was unknown. Such malformations may impair the adult’s ability to mate successfully.

The morphometric changes that larvae undergo during development are critical to normal functioning such as feeding capability and metabolic activity. The sea urchin echinopleuteus larval stage is dependent upon morphological integrity for continued successful development. The ciliated bands located on the larval arms and circumoral surfaces are essential in creating and maintaining feeding currents [167]. Metabolic demands require that the ciliary band length increase in accordance with larval size. The larva accomplishes this by increasing larval arm length and developing additional pairs of larval arms. If the larva’s physical, physiological and metabolic capacity to satisfy these demands are compromised, then the larva will be at a competitive disadvantage – its larval fitness has been compromised.

Lewis *et al.* [74] and Caldwell *et al.* [73] developed a system based on fluctuating developmental asymmetry to precisely quantify very-low-dose sublethal effects of PUA on invertebrate development, thereby providing a useful proxy for larval fitness. Their approach demonstrated harmful effects at PUA concentrations previously deemed safe. The sublethal impacts of PUA during development may be described as altering developmental stability. Developmental stability can be defined as a suite of processes that tend to resist or buffer the disruption of precise development. Developmental instability on the other hand, incorporates a suite of processes that tend to disrupt precise development, such as small differences in rates of cell division, cell growth and cell shape change [168]. A number of techniques have been developed to assess the effects of developmental instability on organisms. Bilaterally symmetrical organisms are regarded as ideal candidates for developmental stability studies. It is accepted that the development of bilaterally symmetric traits are controlled by homologous genes. Therefore the ability of an organism to develop symmetrically while under stress conditions should reflect its propensity to resist environmental or genotoxic stress [169,170] and as such, act as a useful measure of organismal fitness.

The most commonly used descriptor for deviations from perfect symmetry is frequency distributions of right minus left (R-L) trait size. Three general patterns of developmental instability have been described: 1) directional asymmetry which is a pattern of bilateral variation in a sample of individuals, where a statistically significant difference exists between sides, but where the larger side is generally the same; 2) anti-symmetry is a pattern of bilateral variation in a sample of individuals, where a statistically significant difference exists between sides, but where the side that is larger varies at random among individuals; and 3) fluctuating asymmetry is a pattern of bilateral variation in a sample of individuals where the mean of right minus left is zero and variation is normally distributed about that mean [168].

Fluctuating asymmetry studies are based on random fluctuations from the bilaterally symmetrical state of particular characteristics or appendages of an organism [171]. Often fluctuating asymmetry measurements are combined with observations for survival and developmental rate. The measurement of morphological parameters during ontogeny of aquatic organisms is an accepted technique to evaluate the effects of chronic xenobiotic exposure [172] and the use of fluctuating asymmetry measurements as a test of larval fitness is gaining increasing acceptability [173–175].

**Figure 8:**
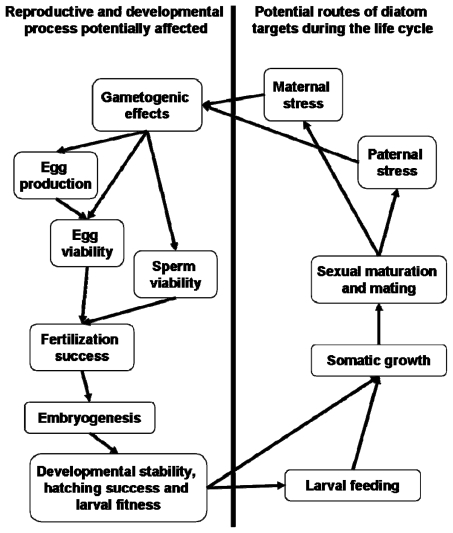
Simplified life cycle of a hypothetical marine invertebrate indicating the potential stages that diatom-oxylipins could interfere with normal ontogenetic processes. For examples refer to [69,71,72,74,92,98,163].

Interestingly, Carotenuto *et al.* [163] suggest that the influence of diatoms on larval fitness is not necessarily limited to developmental instability during embryogenesis. The authors found that healthy copepod nauplii were unable to complete development to adulthood while feeding on diatoms resulting in high mortality. The larvae showed no evidence of anatomical malformations in contrast to nauplii hatched from diatom-fed mothers. These observations suggest that diatoms, aside from being teratogenic, also exert checks on development as a consequence of larval-feeding. In terms of the potential ecological significance, larval copepods therefore must contend with potential embryogenic errors and, assuming survival, also apparent direct toxicity from ingestion (Figure 8). Dinoflagellate controls supported successful development. *C. helgolandicus* nauplii fed *Prorocentrum minimum* with the addition of decadienal were unable to complete development beyond the sixth naupliar stage [5]. This raises the important question of whether diatom-derived toxins can affect somatic growth and hence larval fitness via ingestion by the nauplii.

Clearly, PUA have teratogenic and potentially mutagenic properties [107]. Identifying precisely the point or points during early ontogeny that critical embryonic disturbances occur is difficult given the broad cytotoxic activities of PUA. It would seem, however, that the embryo must cope with attack during significant phases of development. The production of teratogens by plants is common, and indeed the presence of teratogens in vegetables and food-associated microbes has been suggested as the primary reason for morning sickness in human pregnancy. Periods of morning sickness tend to coincide with key stages in embryo development, particularly the formation of the nervous system. Eliminating, or avoiding potentially damaging chemicals from the maternal diet during this phase of embryogenesis would clearly be beneficial. One feature of plant-derived teratogens is that they may otherwise be harmless to the grazer, and indeed the plant source may be highly beneficial for somatic growth, *e.g.,* consumption of *Veratrum californicum* by sheep [176]. However, consumption during pregnancy can result is severe congenital defects. This pattern would seem to hold true for diatom-oxylipins. A diatom-diet appears to support good and reliable copepod somatic growth (however see 163) yet clearly has implications for reproduction. The parallel between terrestrial examples and the diatom-oxylipin scenario would appear to be appropriate.

## 6. Concluding Remarks

The activity of diatom-oxylipins against reproductive and developmental processes is extensive and varied, affecting steps from gametogenesis through to larval competence and the establishment of the larval-body-plan (Figure 8). Despite this broad range of activities it may be possible to rationalise a common molecular and physiological origin. It has already been noted that PUA affect calcium signalling during fertilization [70]. Importantly, the change in intracellular free-calcium concentration is a common intracellular signal in the vast majority of eukaryotic organisms, particularly in relation to reproductive and developmental processes. Aside from the role that calcium plays in the fertilization reaction, it is also functional during embryonic mitoses [177]. Calcium signals have been detected just prior to entry into mitosis and just before initiation of anaphase [178,179]. Interestingly, it is also known that calcium depolymerizes polymerized tubulin *in vitro* [180]. Taken together, these observations are suggestive that the cellular impacts of diatom-PUA may ultimately be traced to interference with intracellular calcium signalling. There are of course other potential mechanisms that may explain the biological responses to PUA including; DNA degradation and transcription errors, decoupling of protein phosphorylation and kinase activity or even simply a loss of essential fatty acids as suggested by Wichard *et al*. [12]. This, of course, remains entirely speculative but verification would seem an appropriate avenue to explore in an attempt to fully understand the activities and ecological functions of this interesting family of biomolecules.

Microorganisms offer an exceptionally rich source of biologically active metabolites and marine microbes in particular are a vastly underexploited resource. There has been growing interest over the last 25 years in microalgal sources of novel compounds with potential pharmacological applications [181]. Diatoms have rarely been screened for biologically active compounds, apart from those expressing antibiotic activity or enzyme inhibition. As such, the production by diatoms of compounds that express such a varied range of reproductive toxicities, and anti-mitotic activities in particular, offers many avenues for future research; both fundamental and applied. Many of the stalwart drugs used in cancer chemotherapy share common properties with diatom-oxylipins including anti-mitotic and teratogenic activities [182]. The search for candidate drugs from marine algae is ripe for expansion; however the traditional bioprospecting approach is both laborious and extremely expensive. An efficiency saving may be found by combining knowledge of algal chemical ecology with pharmacological screening. There may be much to gain by taking a more targeted approach to bioprospecting rather than the traditional shotgun approach. It is important to gain a more thorough and relevant understanding of diatom compounds within a larger ecological and evolutionary framework. To begin to exploit knowledge of marine chemical ecology to inform drug bioprospecting, will require a concerted multidisciplinary approach, studying physiochemical phenomena at the molecular, cellular, organismal, population, and community levels of organization. In this respect, the framework suggested by Moore [183] would seem an effective model to follow. The three key themes to consider are: 1) understanding the mechanisms of molecular and subcellular interactions, including genomic and proteomic aspects; 2) the development of predictive simulation models of effects on complex cellular and physiological processes; and 3) linking molecular, cellular and patho-physiological ‘endpoints’ with higher level ecological consequences. Could ‘chemical ecology guided bioprospecting’ deliver the next generation of marine drugs? Only time will tell.
